# White matter hyperintensities and cerebral microbleeds in persistent post-traumatic headache attributed to mild traumatic brain injury: a magnetic resonance imaging study

**DOI:** 10.1186/s10194-023-01545-w

**Published:** 2023-02-24

**Authors:** Håkan Ashina, Rune H. Christensen, Haidar Muhsen Al-Khazali, Afrim Iljazi, Daniel Tolnai, Anna K. Eigenbrodt, Henrik B. W. Larsson, Henrik W. Schytz, Ulrich Lindberg, Faisal Mohammad Amin

**Affiliations:** 1 Department of Anesthesia, Critical Care and Pain Medicine, Beth Israel Deaconess Medical Center, Harvard Medical School, Boston, MA USA; 2 Department of Neurology, Danish Headache Center, Copenhagen University Hospital - Rigshospitalet, Copenhagen, Denmark; 3 Department of Brain and Spinal Cord Injury, Copenhagen University Hospital - Rigshospitalet, Copenhagen, Denmark; 4 Department of Radiology, Rigshospitalet – Glostrup, Copenhagen, Faculty of Health and Medical Sciences, University of Copenhagen, Copenhagen, Denmark; 5 Functional Imaging Unit, Department of Clinical Physiology, Nuclear Medicine, and PET, Rigshospitalet – Glostrup, Faculty of Health and Medical Sciences, University of Copenhagen, Copenhagen, Denmark

**Keywords:** Neuroimaging, Concussion, Head Trauma, Migraine, Pathophysiology

## Abstract

**Objective:**

To examine whether white matter hyperintensities (WMHs) and cerebral microbleeds (CMBs) are more prevalent in people with persistent post-traumatic headache attributed to mild traumatic brain injury (TBI), compared with healthy controls.

**Methods:**

A magnetic resonance imaging (MRI) study of adults with persistent post-traumatic headache attributed to mild TBI and age- and gender-matched healthy controls. A semi-structured interview and validated self-report instruments were used to record data on demographics, clinical characteristics, and comorbidities. Imaging data were obtained on a 3T MRI Scanner using a 32-channel head coil. Participants and controls underwent a single MRI session, in which fluid-attenuated inversion recovery was used to visualize WMHs, and susceptibility-weighted imaging was used to detect CMBs. The primary outcomes were (I) the difference in the mean number of WMHs between participants with persistent post-traumatic headache and healthy controls and (II) the difference in the mean number of CMBs between participants with persistent post-traumatic headache and healthy controls. All images were examined by a certified neuroradiologist who was blinded to the group status of the participants and controls.

**Results:**

A total of 97 participants with persistent post-traumatic headache and 96 age- and gender-matched healthy controls provided imaging data eligible for analyses. Among 97 participants with persistent post-traumatic headache, 43 (44.3%) participants presented with ≥ 1 WMH, and 3 (3.1%) participants presented with ≥ 1 CMB. Compared with controls, no differences were found in the mean number of WMHs (2.7 vs. 2.1, *P* = 0.58) and the mean number of CMBs (0.03 vs. 0.04, *P* = 0.98).

**Conclusions:**

WMHs and CMBs were not more prevalent in people with persistent post-traumatic headache than observed in healthy controls. Future studies should focus on other MRI techniques to identify radiologic biomarkers of post-traumatic headache.

## Introduction

Post-traumatic headache is a disabling neurologic disorder, which is most often attributed to mild traumatic brain injury (TBI), [1]. Although the disorder tends to remit spontaneously in most people, some experience persistence of headache which can last years and resists all varieties of treatment [1–5].

The neurobiologic basis of post-traumatic headache is not fully understood [6], but magnetic resonance imaging (MRI) is increasingly being used to investigate whether structural lesions might be related to sequelae of mild TBI, including headache [7, 8]. The reported associations of mild TBI with white matter hyperintensities (WMHs) and cerebral microbleeds (CMBs) have raised questions about the significance of these findings [9–11], and whether they are related to the genesis of cephalic pain after head trauma. A growing body of evidence is being marshalled in support of this assertion, and one recent MRI study showed findings suggestive of increased iron accumulation in people with acute post-traumatic headache, compared with age-matched healthy controls [12]. In this context, the iron accumulation might indicate the presence of CMBs. The latter is best evaluated using susceptibility-weighted imaging (SWI), which is superior to conventional T2*-weighted gradient-recalled echo imaging in detecting CMBs [13–15]. There is however no data on the extent of WMHs and CMBs in people with persistent post-traumatic headache. As such, it seems timely to explore if any such relationship exists which, in turn, might establish the first radiologic biomarkers of persistent post-traumatic headache.

In this cross-sectional MRI study, we examine whether WMHs and CMBs are more prevalent in 97 people with persistent post-traumatic headache attributed to mild TBI, compared with 96 age- and gender-matched healthy controls. To this end, we used the fluid-attenuated inversion recovery (FLAIR) sequence to visualize WMHs and SWI to detect CMBs.

## Methods

### Study oversight

The study protocol was approved by the relevant ethics committee, and the study was conducted in adherence to the principles of the Declaration of Helsinki. All participants provided written informed consent before any study-related procedures or tasks were performed. A group of site investigators (H.A., R.H.C., and F.M.A.) reviewed the acquired data, and all authors vouch for the accuracy and completeness of the data. The first and senior author had unrestricted data access and drafted the initial version of the manuscript, which was revised for important intellectual content by all authors.

### Study population

Participants and healthy controls were identified and enrolled with previous published methods [16, 17]. Eligible participants were aged 18 to 65 years and had a diagnosis of persistent post-traumatic headache in accordance with the 3^rd^ edition of the International Classification of Headache Disorders (ICHD-3), [18]. Another inclusion criterion was that mild TBI had to have occurred at least 12 months before enrollment. Participants were excluded if they had a history of more than one TBI as well as any history of whiplash injury, medication-overuse headache, or a primary headache disorder, except for infrequent episodic tension-type headache (TTH). Contraindications to MRI were also considered reasons for exclusion. Detailed eligibility criteria have been published elsewhere [19].

Healthy controls were eligible for inclusion if they were aged 18 to 65 years and had no history of TBI, whiplash injury, primary headache disorder (except for infrequent episodic TTH), neurologic or psychiatric disorders, and cardiovascular disease. Controls were also excluded if they had first-degree relatives with any primary headache disorder or reported daily intake of any medication other than oral contraceptives.

### Study design and procedures

The present study had a cross-sectional design and was conducted between July 2018 and June 2019 at a single center. Data were collected using a semi-structured interview and included information on demographics, clinical characteristics, comorbidities, and medication history. Additional data on the presence of comorbid anxiety, depression, and mild cognitive impairment was collected using validated self-report instruments, as detailed elsewhere [17]. The Hospital Anxiety and Depression Scale (HADS) was used to screen for anxiety and depression separately in both participants and controls [16], whereas the Montreal Cognitive Assessment (MoCA) questionnaire was used to screen for mild cognitive impairment in participants only [20]. In addition, the Pittsburg Sleep Quality Index (PSQ-I) was used to evaluate quality of sleep in both participants and controls [21]. Imaging data were obtained on a 3T MRI Scanner (Philips Achieva) using a 32-channel head coil. Participants and controls underwent a single MRI session with a standardized protocol that included 3D T1-weighted images, SWI, and T2-weighted FLAIR.

### Image analysis

All images were transferred to a centralized server, where they were examined by a certified neuroradiologist (D.T.) who was blinded to the group status of the participants and controls. Images were interpreted for the presence, number, and location of WMHs on FLAIR and CMBs on SWI. WMHs were defined as hyperintense lesions that were ≥ 2 mm and visible on FLAIR, whereas CMBs were defined as punctate or ovoid lesions visible on SWI. The Fazekas scale was used to rate the size and confluence of exclusively periventricular WMHs on a 4-point Likert scale, with a score of 0 indicating absence of WMHs while a score of 3 denotes pronounced white matter pathology. More details on the Fazekas scale have been published elsewhere [22]. Pathologic findings and lesions that were poorly contrasted (e.g., due to motion artifacts or bad image quality) were excluded from the final analysis. All detected anatomical variants and structural abnormalities were recorded, and incidental findings of clinical relevance were elaborated in a text box which allowed the neuroradiologist to provide guidance on recommended clinical and/or imaging follow-up. The resulting report was sent to the senior author (F.M.A.) who was then responsible for initiation of appropriate follow-up. Fifteen scans were excluded due to insufficient image quality or pathologic findings requiring clinical referral.

### Outcomes

The primary outcomes were (I) the difference in the mean number of WMHs between participants with persistent post-traumatic headache and healthy controls and (II) the difference in the mean number of CMBs between participants with persistent post-traumatic headache and healthy controls. The secondary outcomes were (I) the difference in the number of individuals with ≥ 1 WMHs between participants and controls and (II) the difference in the number of individuals with ≥ 1 CMBs between participants and controls.

### Statistical analysis

Descriptive statistics were used to summarize demographics and clinical characteristics. Normal distribution was assessed visually and using the Shapiro–Wilk test. Continuous data were presented with means and standard deviations (SDs) when normally distributed and with medians and interquartile ranges (IQR) when not normally distributed. Categorical data was summarized as proportions (or percentages). The Mann–Whitney U test was used to compare the mean number of WMHs and CMBs between participants with persistent post-traumatic headache and healthy controls. The Fisher’s exact test was used for comparisons of the number of individuals with ≥ 1 WMHs and the number individuals with ≥ 1 CBMs between participants and controls. Correlation coefficients, *ρ*, were calculated using the Spearman rank test. The level of significance was set at *P* < 0.05 (2-sided), and no corrections were made for multiple comparisons.

## Results

**Fig. 1:**
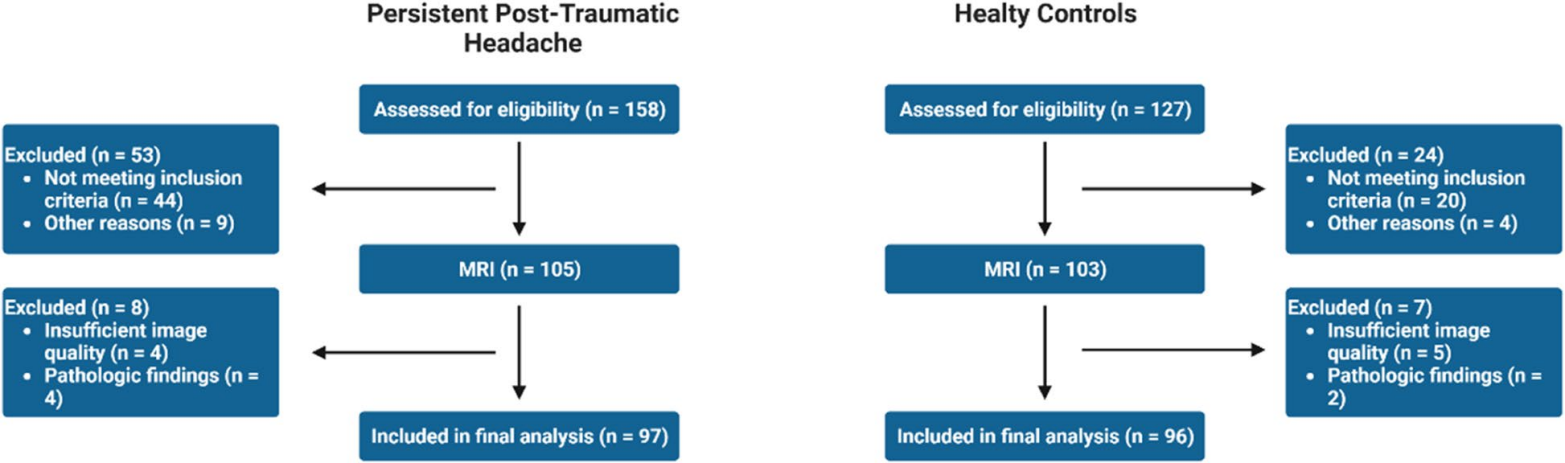
Flow of Participant Enrollment

A total of 97 enrolled participants with persistent post-traumatic headache and 96 age- and gender-matched healthy controls provided imaging data on WMHs and CMBs that were deemed eligible for analyses (Fig. 1). Fifteen scans were excluded due to insufficient image quality or pathologic findings that required clinical referral.

Participants’ mean age was 35.8 ± 11.6 years, and 80 (82.5%) were women. Age and gender were comparable between participants and controls (Table 1). In addition, participants reported a mean headache frequency of 25.5 ± 7.1 days per month, and most participants had a migraine-like phenotype (88 [90.8%] of 97). The clinical characteristics of the participants are described in Table 2.

**Table 1:** Summary of the Study Populations

Variable	Participants with Persistent Post-Traumatic Headache (*n* = 97)	Healthy Controls (*n* = 96)	Z-scores	*P* Values
**Age**, mean (SD), y	35.8 (11.6)	35.9 (11.4)	0.07	0.95
**Male/Female**, n	17/80	17/79	-	1.00
**Height**, mean (SD), cm	171.6 (8.0)	171.2 (8.8)	0.59	0.55
**Weight**, mean (SD), kg	72.4 (14.5)	71.7 (14.2)	0.59	0.56
**BMI**, mean (SD), kg/m^2^	24.5 (4.2)	24.4 (3.7)	0.53	0.59
**WMHs**,				
Mean (SD)	2.7 (7.1)	2.1 (3.9)	0.56	0.58
No WMHs, n (%)	54 (55.7)	48 (50.0)		-
≥ 1 WMH, n (%)	43 (44.3)	48 (50.0)		0.47
≥ 2 WMHs, n (%)	30 (30.9)	34 (35.1)		-
≥ 3 WMHs, n (%)	20 (20.6)	20 (20.8)		-
**CMBs**,				
Mean (SD)	0.03 (0.2)	0.04 (0.2)	0.02	0.98
No CMBs, n (%)	94 (96.9)	93 (96.9)		-
≥ 1 CMB, n (%)	3 (3.1)	3 (3.1)		1.00
2 CMBs, n (%)	0 (0.0)	1 (1.0)		-

*SD* Standard deviation, *BMI* Body Mass Index, *WMH* White matter hyperintensities, *CMB* Cerebral microbleeds

**Table 2:** Characteristics of the Post-Traumatic Headache Population

Characteristics	Persistent Post-Traumatic Headache (*n* = 97)
**Disease Duration**, median (IQR), months	37 (23–66)
**Headache Phenotypes**, n (%)	
Chronic migraine-like	60 (61.9)
Episodic migraine-like	1 (1.0)
Episodic migraine-like combined with chronic TTH-like	23 (23.7)
Episodic migraine-like combined with frequent TTH-like	2 (2.1)
Episodic migraine-like combined with infrequent TTH-like	2 (2.1)
Chronic TTH-like	9 (9.3)
**Headache Frequency**, mean (SD)	
Monthly headache days	25.5 (7.1)
**Current Use of Preventive Medications**, n (%)	61 (62.9)
**HADS Anxiety Scores**, mean score (SD)	8.4 (4.6)
Probable or high risk of anxiety, %	51 (52.6)
Probable risk of anxiety, %	19 (19.8)
High risk of anxiety, %	32 (33.3)
**HADS Depression Scores**, mean score (SD)	6.7 (3.9)
Probable or high risk of depression, n (%)	42 (43.3)
Probable risk of depression, n (%)	30 (32.0)
High risk of depression, n (%)	12 (12.4)
**MoCA**, mean score (SD)	26.3 (2.3)
Mild cognitive impairment, n (%)	19 (19.6)
**Global PSQ-I Score,** mean score (SD)	8.9 (3.9)
Poor quality of sleep, n (%)	83 (85.6)

*IQR* Interquartile range, *SD* Standard deviation, *HADS* Hospital Anxiety and Depression Scale, *MoCA* Montreal Cognitive Assessment, *PSQ-I* Pittsburg Sleep Quality Index

### White matter hyperintensities

**Fig. 2:**
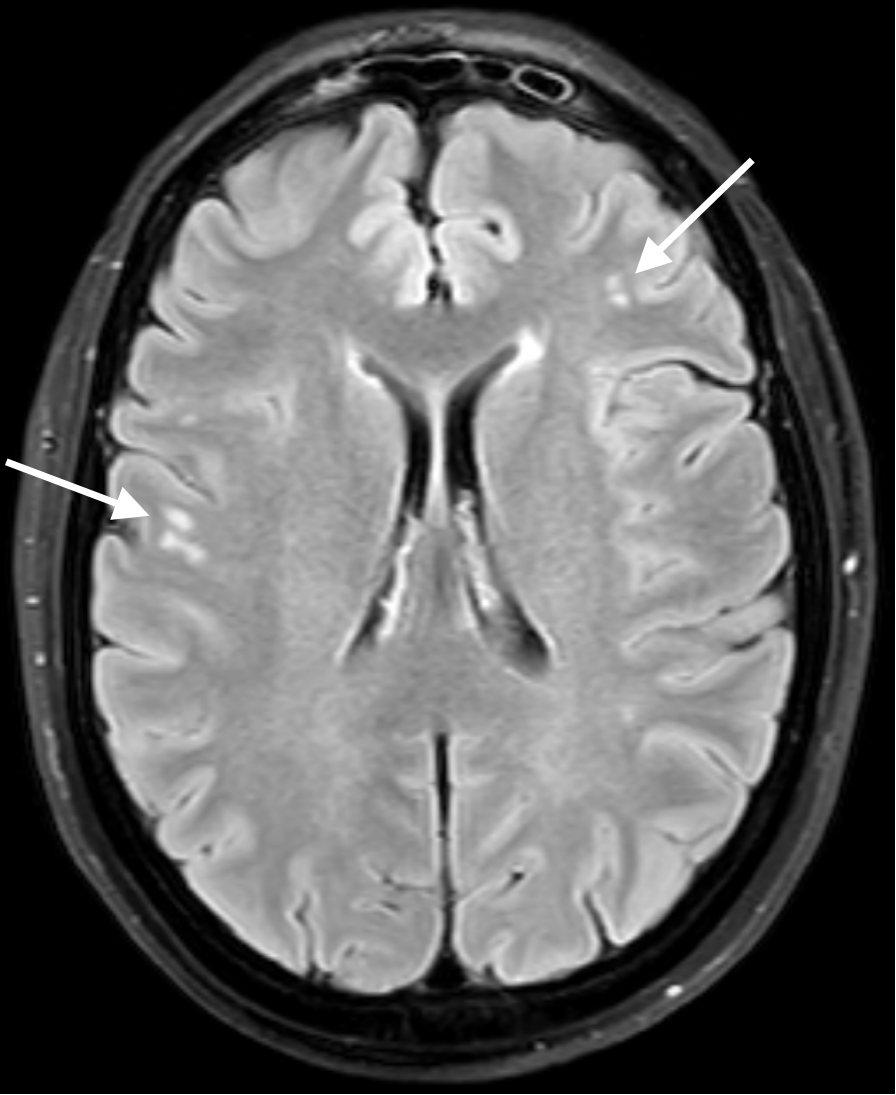
Example of white matter hyperintensities

Among 97 participants with persistent post-traumatic headache, 43 (44.3%) presented with ≥ 1 WMH, 30 (30.9%) had ≥ 2 WMHs, and 20 (20.6%) had ≥ 3 WMHs (Table 1, Fig. 2). No WMHs were observed in the other 54 (55.7%) participants. There was no difference in the mean number of WMHs between participants with persistent post-traumatic headache and age- and gender-matched healthy controls (2.7 vs. 2.1, *P* = 0.58; Table 1). We found no difference in the number of individuals with ≥ 1 WMHs between participants and controls (43 vs. 48, *P* = 0.47). Exploratory analyses revealed no correlations of number of WMHs with HADS anxiety scores, HADS depression scores, MoCA scores, global PSQ-I score, or headache intensity on the MRI scan day (Table 3).

**Table 3:** Correlations of White Matter Hyperintensities with Anxiety, Depression, Mild Cognitive Impairment, Poor Quality of Sleep, Headache Intensity, and Disease Duration in Persistent Post-Traumatic Headache (*n* = 97)

Variable	Correlation Coefficient, *ρ*	*P* value
HADS Anxiety Scores	< 0.01	0.99
HADS Depression Scores	0.14	0.17
MoCA Scores	-0.10	0.35
Global PSQ-I Scores	0.11	0.30
Headache Intensity^a^	-0.17	0.10
Disease Duration^b^	0.13	0.20

*HADS* Hospital Anxiety and Depression Scale, *MoCA* Montreal Cognitive Assessment, *PSQ-I* Pittsburg Sleep Quality Index

^a^ Headache intensity was measured on an 11-point numeric rating scale (NRS, 0 being no headache, 10 being the worst imaginable headache)

^b^ Disease duration was recorded as the number of months since mild traumatic brain injury

### Cerebral microbleeds

**Fig. 3:**
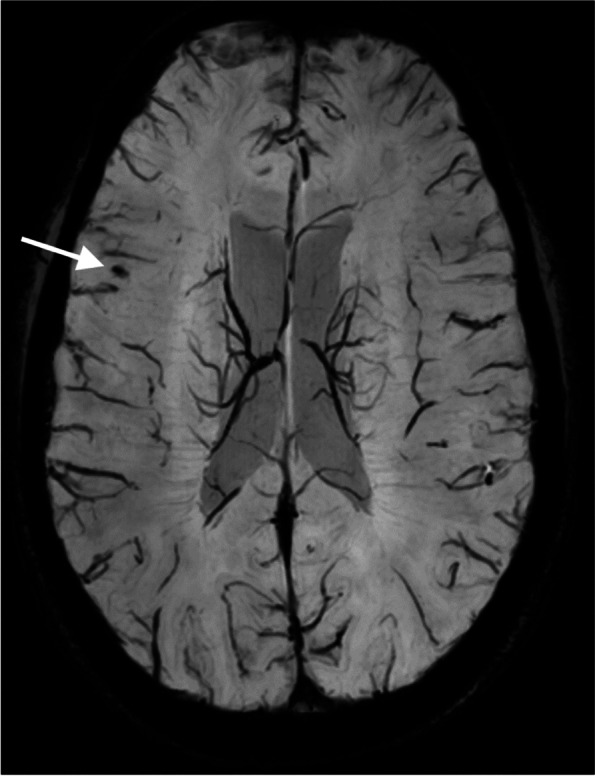
Example of a Cerebral Microbleed

Among 97 participants with persistent post-traumatic headache, 3 (3.1%) presented with 1 CMB. No CMBs were found in the other 94 (96.9%) participants (Table 1, Fig. 3). The mean number of CMBs did not differ between participants with persistent post-traumatic headache and age- and gender-matched healthy controls (0.3 vs. 0.4, *P* = 0.98). In addition, we found no difference in the number of individuals with ≥ 1 CMBs between participants and controls (3 vs. 3 *P* = 1.00).

## Discussion

The main findings of this study were that WMHs and CMBs are not more prevalent in people with persistent post-traumatic headache, compared with age- and gender-matched healthy controls. To our knowledge, the present study is the first to assess the associations of persistent post-traumatic headache with WMHs and CMBs.

From a clinical perspective, it is interesting to know the prevalence of WMHs and CMBs in people with persistent post-traumatic headache, as this can help contextualize the importance of similar findings on clinical imaging. However, the present data did not support any association of persistent post-traumatic headache with WMHs and CBMs. This aligns well with some previous reports that included people with mild TBI or post-concussive sequelae [23, 24]. For example, one MRI study scanned 45 ice hockey players at the beginning and the end of the hockey season [23]. The authors found no significant changes in the number of WMHs and CMBs attributable to concussion when comparing baseline with follow-up scans. In line with this observation, another MRI study reported no differences between 127 participants with post-concussion syndrome and 29 age- and sex-matched controls in terms of WMHs and CMBs [24]. Among the 127 participants, WMHs were observed in 28 (22.0%) participants, and CMBs were detectable only in 2 (1.6%) participants. It does, nonetheless, merit emphasis that there is conflicting MRI data on whether mild TBI is associated with WMHs and CMBs [9–11]. Indeed, one MRI study reported that CMBs were present in 26 (23.4%) of 111 patients with mild TBI, compared with 12 (10.8%) of 111 controls [10]. The authors used susceptibility-weighted angiography to detect CMBs, whereas the present study used SWI. There is, to our knowledge, no robust evidence of susceptibility-weighted angiography being superior to SWI in detecting CMBs. In another MRI study [25], the authors found no evidence of mild TBI being associated with CMBs, as only 29 (3.5%) of 768 participants had detectable CMBs. Interestingly, the same study reported that 432 (51.8%) of 834 participants with predominantly mild TBI had WMHs, compared with 16 (38.1%) of 42 controls [26]. Taken together, there is no firm evidence to support an association of mild TBI or its sequelae with WMHs and CMBs that can contextualize findings on clinical imaging.

The incongruent prevalence rates of WMHs and CMBs across MRI studies might be attributed to differences in subject selection and methods used. Several studies have established that the number of WMHs and CMBs increases with older age [26–28], and CMBs are furthermore associated with hypertension [27]. There is also MRI data to support that the number and volume of CMBs decrease over time in military service members with TBI [29]. Thus, it cannot be excluded that CMBs might be pathogenic drivers of post-traumatic headache in the acute phase. In fact, recent MRI data showed increased iron accumulation in specific brain regions of 20 people with acute post-traumatic headache, compared with 20 healthy controls [12]. Iron accumulation might reflect CMBs, but more evidence is needed to examine adequately whether acute post-traumatic headache is associated with CMBs. On a different note, the choice of MRI sequence is also important in detecting CMBs, as SWI has been shown to be more sensitive than conventional gradient-recalled echo imaging [30].

### Limitations

This study has several limitations. First, our cross-sectional design does not allow for causality to be inferred. In regard to CMBs, we can also not exclude a decrease in number and volume since the mild TBI occurred. Nonetheless, it seems less likely that CMBs are of pathophysiologic importance, as we observed them in only 3 (3.1%) of 97 participants with persistent post-traumatic headache. Second, participants were identified mainly from the outpatient clinic of the Danish Headache Center. The study population is thus not representative of people with persistent post-traumatic headache in the general population, as the present study population is probably more adversely affected. Third, the present study included predominantly female participants, and we can therefore not exclude that WMHs and CMBs have pathophysiologic importance in males with persistent post-traumatic headache. Fourth, WMHs and CMBs were quantified by only one board-certified neuroradiologist. This approach might be prone to errors, and verification from other neuroradiologist should be considered in future investigations. Lastly, we cannot exclude that the number of CMBs were underestimated, as imaging techniques are so far inferior to histopathologic analysis [30].

## Conclusions

Among people with persistent post-traumatic headache, WMHs and CMBs are not more prevalent than observed in age- and gender-matched healthy controls free of headache and TBI. Future MRI studies should focus on identifying radiological biomarkers of persistent post-traumatic headache using diffusion, functional, or metabolic imaging.
